# Crystal structure of {(*E*)-2-[(phenyl­imino)­meth­yl]phenolato-κ^2^ *N*,*O*}bis­[2-(pyridin-2-yl)phenyl-κ^2^ *C* ^1^,*N*]iridium(III) di­chloro­methane monosolvate

**DOI:** 10.1107/S2056989016008100

**Published:** 2016-05-20

**Authors:** Moo-Sung Goo, Ki-Min Park, Hee-Joon Kim

**Affiliations:** a Department of Applied Chemistry, Kumoh National Institute of Technology, Gumi 39177, Republic of Korea; b Research institute of Natural Science, Gyeongsang National University, Jinju 52828, Republic of Korea

**Keywords:** crystal structure, iridium(III) complex, C_2_N_3_O coordination set

## Abstract

In the title compound, [Ir(C_11_H_8_N)_2_(C_13_H_10_NO)]·CH_2_Cl_2_, the Ir^III^ ion is six-coordinated by two *C*,*N*-bidentate 2-(pyridin-2-yl)phenyl ligands and one *N*,*O*-bidentate 2-[(phenyl­imino)­meth­yl]phenolate anion, giving rise to a distorted octa­hedral environment. The *C*,*N*-bidentate ligands, in which the C and N atoms are statistically disordered over two sites and therefore both pairs of C and N atoms are *trans* and *cis* relative to each other, are almost perpendicular to each other [the dihedral angle between the least-square planes is 87.00 (4)°]. An intra­molecular C—H⋯O hydrogen bond, as well as inter­molecular C—H⋯π inter­actions and π–π inter­actions, contribute to the stabilization of the mol­ecular and crystal structure.

## Abstract 2

The Ir^III^ atom in the title mol­ecule adopts a distorted octa­hedral coordination sphere, being *C*,*N*-chelated by two 2-(pyridin-2-yl)phenyl ligands and *N*,*O*-chelated by one ancillary 2-[(phenyl­imino)­meth­yl]phenolate ligand. The crystal packing is stabilized by inter­molecular C—H⋯π inter­actions and π–π inter­actions.

## Chemical context   

Cyclo­metallated Ir^III^ complexes are of great inter­est due to their excellent phospho­rescent properties and electroluminescence applications. In particular, heteroleptic Ir^III^ complexes with imine-based ancillary ligands exhibit aggregation-induced phospho­rescent emission (AIPE), resulting in enhanced phospho­rescence phenomena in the solid state (Howarth *et al.*, 2014 ▸; You *et al.*, 2008 ▸; Zhao *et al.*, 2008 ▸). To uncover the origin of the intriguing AIPE, it is crucial to analyse the solid-state structures of relevant Ir^III^ complexes besides undertaking spectroscopic and theoretical investigations. Here we report the crystal structure of the title compound, [Ir(C_11_H_8_N)_2_(C_13_H_10_NO)]·CH_2_Cl_2_, a heteroleptic Ir^III^ complex with an ancillary salicyl­imine ligand.
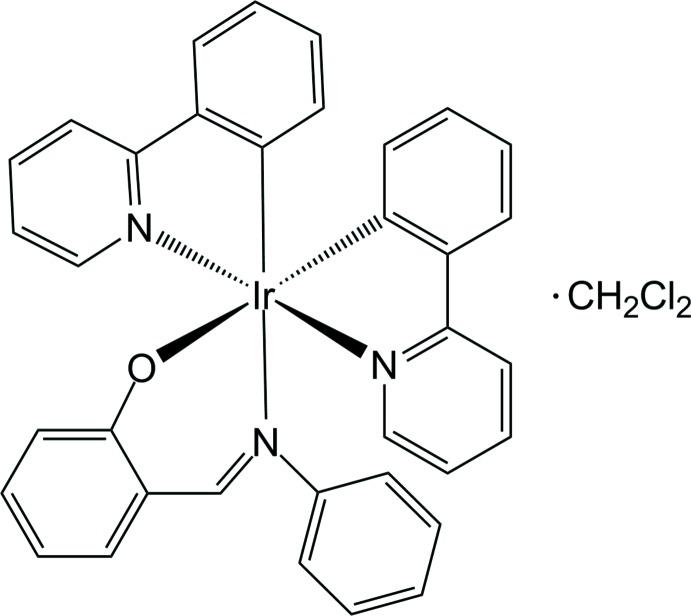


## Structural commentary   

**Figure 1:**
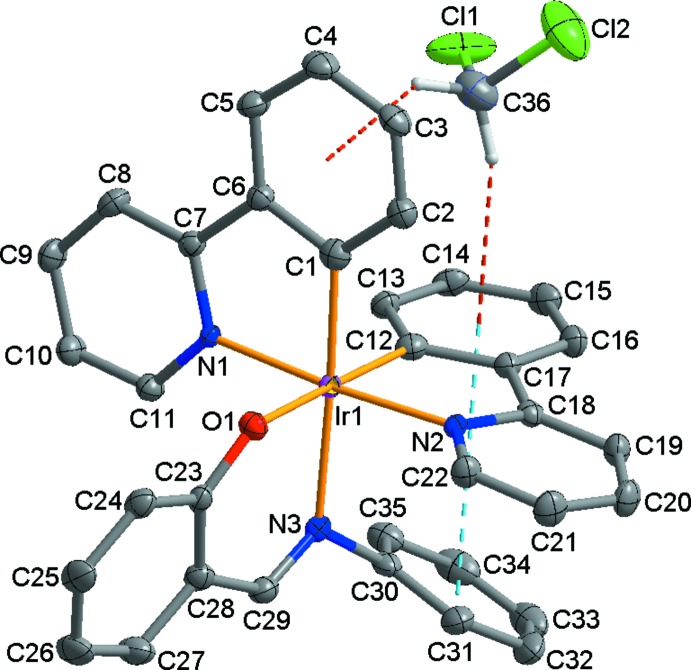
View of the mol­ecular structure of the title compound, showing the atom-numbering scheme. Displacement ellipsoids are drawn at the 50% probability level; red and sky-blue dashed lines represent inter­molecular C—H⋯π hydrogen bonds and intra­molecular π–π inter­actions, respectively. H atoms have been omitted for clarity.

The mol­ecular components of the title structure are shown in Fig. 1 ▸. The asymmetric unit consists of one Ir^III^ ion, two 2-(pyridin-2-yl)phenyl ligands, and one 2-[(phenyl­imino)­meth­yl]phenolate anion. The Ir^III^ ion adopts a distorted octa­hedral coordination geometry, being *N*,*O*-chelated by the 2-[(phenyl­imino)­meth­yl]phenolate ligand and *C*,*N*-chelated by two 2-(pyridin-2-yl)phenyl ligands, in which the C and N atoms are equally disordered over two sites and therefore both pairs of C and N atoms are *trans* and *cis* relative to each other. The equatorial plane is formed by N1/O1/N2/C12 atoms, the mean deviation from the least-squares plane being 0.002 Å. The Ir^III^ ion is displaced by 0.0481 (9) Å from the equatorial plane towards the axial imino N3 atom. The *C*,*N*-bidentate ligands are nearly perpendicular to each other, with a dihedral angle between the least-squares planes of 87.00 (4)°. Within the *C*,*N*-bidentate ligands, the dihedral angles between the aromatic rings are 3.70 (10) (between rings C1–C6 and N1/C7–C11) and 7.67 (16)° (between rings C12–C17 and N2/C18–C22). As shown in Table 1 ▸, the Ir—C, Ir—N and Ir—O bond lengths of the title compound are within the ranges reported for similar Ir^III^ compounds, *e.g*. {(*E*)-2-[(2,6-diiso­propyl­phenyl­imino)­meth­yl]phenolato-*κ* ^2^ *N*,*O*}bis­(2-phenyl­pyridine-*κ* ^2^ *C*,*N*)iridium(III) (Howarth *et al.*, 2014 ▸), {(*E*)-2-[(naphthalene-1-yl­imino)­meth­yl]phenolato-*κ* ^2^ *N*,*O*}bis­(2-phenyl­pyridine-*κ* ^2^ *C*,*N*)iridium(III) (Zhao *et al.*, 2008 ▸), or {(*E*)-2-[(phenyl­imino)­meth­yl]phenolato-*κ* ^2^ *N*,*O*}bis­[2-(2,4-di­fluoro­phen­yl)pyridine*-κ* ^2^ *C*,*N*]iridium(III) (You *et al.*, 2008 ▸).

## Supra­molecular features   

**Figure 2:**
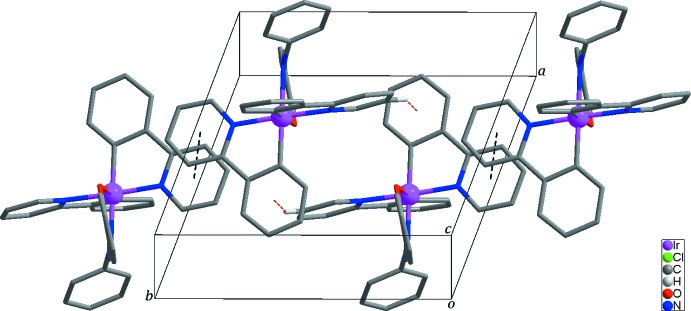
Packing plot of the mol­ecular components in the title compound. Yellow and black dashed lines represent inter­molecular C—H⋯π and π–π stacking inter­actions, respectively. H atoms not involved in inter­molecular inter­actions and di­chloro­methane solvent mol­ecules have been omitted for clarity.

The mol­ecular structure of the title compound is stabilized by an intra­molecular C—H⋯O hydrogen bond and inter­molecular C—H⋯π inter­actions between the di­chloro­methane solvent mol­ecule and the phenyl rings of the *C*,*N*-bidentate ligand (Fig. 1 ▸ and Table 2 ▸). Additionally, inter­molecular C—H⋯π inter­actions (Table 2 ▸) and π–π inter­actions [*Cg*1⋯*Cg*1^ii^ = 3.6231 (12) Å and *Cg*3⋯*Cg*4 = 3.8873 (17) Å; *Cg*1, *Cg*3 and *Cg*4 are the centroids of the N1/C7–C11, C12–C17 and C30–C35 rings, respectively; symmetry code: (ii) −*x* + 1, −*y* + 2, −*z* + 1] contribute to the stabilization of the crystal structure (Fig. 2 ▸).

## Synthesis and crystallization   

The title compound was prepared according to a reported procedure (You *et al.*, 2008 ▸). Single crystals suitable for X-ray diffraction were grown by slow diffusion of *n*-hexane into a CH_2_Cl_2_ solution of the title compound at room temperature.

## Refinement   

Crystal data, data collection and structure refinement details are summarized in Table 3 ▸. The positions of the N atoms in the 2-(pyridin-2-yl)phenyl unit could not be discriminated from the difference in the displacement parameters, and free refinement of the N and C atoms revealed a lower and higher electron density, respectively, as expected for full occupancy and without disorder. Therefore, atoms N1 and C1*A*, C11 and N1*A*, N2 and C2*A*, and C22 and N2*A* were refined at the same sites with site occupancy factors of 0.5 using EXYZ/EADP constrains. All H atoms were positioned geometrically and refined using a riding model, with C—H = 0.95 Å for C*sp* ^2^—H and 0.99 Å for methyl­ene C—H. For all H atoms, *U*_iso_(H) = 1.2*U*_eq_ of the parent atom.

## Supplementary Material

Crystal structure: contains datablock(s) I, New_Global_Publ_Block. DOI: 10.1107/S2056989016008100/wm5290sup1.cif

Structure factors: contains datablock(s) I. DOI: 10.1107/S2056989016008100/wm5290Isup2.hkl

CCDC reference: 1480710

Additional supporting information:  crystallographic information; 3D view; checkCIF report

## Figures and Tables

**Table 1:** Selected geometric parameters (Å, °)

Ir1—C12	1.997 (2)	Ir1—N1	2.0424 (18)
Ir1—C1	2.004 (2)	Ir1—O1	2.1409 (16)
Ir1—N2	2.0302 (18)	Ir1—N3	2.1551 (19)
			
C12—Ir1—C1	87.97 (9)	N2—Ir1—O1	94.95 (7)
C12—Ir1—N2	80.69 (8)	N1—Ir1—O1	89.08 (7)
C1—Ir1—N2	96.62 (8)	C12—Ir1—N3	94.96 (8)
C12—Ir1—N1	95.15 (8)	C1—Ir1—N3	176.86 (7)
C1—Ir1—N1	80.42 (8)	N2—Ir1—N3	84.99 (7)
N2—Ir1—N1	175.02 (7)	N1—Ir1—N3	98.16 (7)
C12—Ir1—O1	175.01 (7)	O1—Ir1—N3	87.04 (7)
C1—Ir1—O1	90.14 (8)		

**Table 2:** Hydrogen-bond geometry (Å, °) *Cg*2 and *Cg*3 are the centroids of the C1–C6 and C12–C17 rings, respectively.

*D*—H⋯*A*	*D*—H	H⋯*A*	*D*⋯*A*	*D*—H⋯*A*
C22—H22⋯O1	0.95	2.54	3.132 (3)	121
C21—H21⋯*Cg*2^i^	0.95	2.90	3.658 (3)	138
C36—H36*A*⋯*Cg*2	0.99	2.62	3.444 (4)	140
C36—H36*B*⋯*Cg*3	0.99	2.59	3.498 (4)	153

Symmetry code: (i) 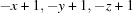.

**Table 3:** Experimental details

Crystal data	
Chemical formula	[Ir(C_11_H_8_N)_2_(C_13_H_10_NO)]·CH_2_Cl_2_
*M*_r_	781.71
Crystal system, space group	Triclinic, *P* 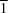
Temperature (K)	130
*a*, *b*, *c* (Å)	11.8318 (2), 12.0638 (4), 12.3169 (2)
α, β, γ (°)	98.260 (1), 114.283 (1), 101.418 (1)
*V* (Å^3^)	1520.15 (6)
*Z*	2
Radiation type	Mo *K*α
μ (mm^−1^)	4.60
Crystal size (mm)	0.15 × 0.09 × 0.05

Data collection	
Diffractometer	Bruker APEXII CCD
Absorption correction	Multi-scan (*SADABS*; Bruker, 2013 ▸)
*T*_min_, *T*_max_	0.614, 0.877
No. of measured, independent and observed [*I* > 2σ(*I*)] reflections	25306, 7425, 6973
*R*_int_	0.020
(sin θ/λ)_max_ (Å^−1^)	0.666

Refinement	
*R*[*F* ^2^ > 2σ(*F* ^2^)], *wR*(*F* ^2^), *S*	0.019, 0.047, 1.08
No. of reflections	7425
No. of parameters	388
H-atom treatment	H-atom parameters constrained
Δρ_max_, Δρ_min_ (e Å^−3^)	1.45, −1.25

Computer programs: *APEX2* and *SAINT* (Bruker, 2013 ▸), *SHELXS97* and *SHELXTL* (Sheldrick, 2008 ▸), *SHELXL2014* (Sheldrick, 2015 ▸) and *DIAMOND* (Brandenburg, 2010 ▸).

## supplementary crystallographic information

### Crystal data

**Table:**

[Ir(C_11_H_8_N)_2_(C_13_H_10_NO)]·CH_2_Cl_2_	*Z* = 2
*M**_r_* = 781.71	*F*(000) = 768
Triclinic, *P*1	*D*_x_ = 1.708 Mg m^−^^3^
*a* = 11.8318 (2) Å	Mo *K*α radiation, λ = 0.71073 Å
*b* = 12.0638 (4) Å	Cell parameters from 25306 reflections
*c* = 12.3169 (2) Å	θ = 1.8–28.3°
α = 98.260 (1)°	µ = 4.60 mm^−^^1^
β = 114.283 (1)°	*T* = 130 K
γ = 101.418 (1)°	Plate, yellow
*V* = 1520.15 (6) Å^3^	0.15 × 0.09 × 0.05 mm

### Data collection

**Table:**

Bruker APEXII CCD diffractometer	6973 reflections with *I* > 2σ(*I*)
φ and ω scans	*R*_int_ = 0.020
Absorption correction: multi-scan (*SADABS*; Bruker, 2013)	θ_max_ = 28.3°, θ_min_ = 1.8°
*T*_min_ = 0.614, *T*_max_ = 0.877	*h* = −15→15
25306 measured reflections	*k* = −16→16
7425 independent reflections	*l* = −15→16

### Refinement

**Table:**

Refinement on *F*^2^	0 restraints
Least-squares matrix: full	Hydrogen site location: inferred from neighbouring sites
*R*[*F*^2^ > 2σ(*F*^2^)] = 0.019	H-atom parameters constrained
*wR*(*F*^2^) = 0.047	*w* = 1/[σ^2^(*F*_o_^2^) + (0.0286*P*)^2^ + 0.6203*P*] where *P* = (*F*_o_^2^ + 2*F*_c_^2^)/3
*S* = 1.08	(Δ/σ)_max_ = 0.003
7425 reflections	Δρ_max_ = 1.45 e Å^−^^3^
388 parameters	Δρ_min_ = −1.25 e Å^−^^3^

### Special details

**Table:**

Geometry. All esds (except the esd in the dihedral angle between two l.s. planes) are estimated using the full covariance matrix. The cell esds are taken into account individually in the estimation of esds in distances, angles and torsion angles; correlations between esds in cell parameters are only used when they are defined by crystal symmetry. An approximate (isotropic) treatment of cell esds is used for estimating esds involving l.s. planes.

### Fractional atomic coordinates and isotropic or equivalent isotropic displacement parameters (Å^2^)

**Table:**

	*x*	*y*	*z*	*U*_iso_*/*U*_eq_	Occ. (<1)
Ir1	0.61255 (2)	0.74630 (2)	0.71617 (2)	0.01224 (3)	
Cl1	0.23841 (8)	0.81561 (9)	0.92075 (9)	0.0577 (2)	
Cl2	0.13261 (10)	0.57002 (9)	0.79287 (9)	0.0624 (3)	
C36	0.2462 (3)	0.7010 (3)	0.8194 (3)	0.0384 (7)	
H36A	0.2294	0.7215	0.7402	0.046*	
H36B	0.3341	0.6908	0.8553	0.046*	
O1	0.62551 (15)	0.70735 (14)	0.54724 (15)	0.0181 (3)	
N1	0.58415 (18)	0.90242 (16)	0.68412 (17)	0.0112 (4)	0.5
C1A	0.58415 (18)	0.90242 (16)	0.68412 (17)	0.0112 (4)	0.5
N2	0.62991 (18)	0.59058 (16)	0.75448 (18)	0.0118 (4)	0.5
C2A	0.62991 (18)	0.59058 (16)	0.75448 (18)	0.0118 (4)	0.5
N3	0.82100 (18)	0.80078 (16)	0.80795 (17)	0.0152 (4)	
C1	0.4191 (2)	0.69766 (19)	0.6226 (2)	0.0212 (4)	0.5
N1A	0.4191 (2)	0.69766 (19)	0.6226 (2)	0.0212 (4)	0.5
C2	0.3326 (2)	0.5863 (2)	0.5879 (2)	0.0195 (5)	
H2	0.3656	0.5232	0.6112	0.023*	
C3	0.1996 (2)	0.5657 (2)	0.5204 (2)	0.0233 (5)	
H3	0.1437	0.4889	0.4971	0.028*	
C4	0.1480 (2)	0.6570 (2)	0.4865 (2)	0.0261 (5)	
H4	0.0572	0.6428	0.4407	0.031*	
C5	0.2310 (2)	0.7689 (2)	0.5207 (2)	0.0226 (5)	
H5	0.1968	0.8317	0.4986	0.027*	
C6	0.3646 (2)	0.78903 (19)	0.5875 (2)	0.0169 (4)	
C7	0.4586 (2)	0.90398 (19)	0.6250 (2)	0.0162 (4)	
C8	0.4294 (2)	1.0079 (2)	0.6042 (2)	0.0209 (5)	
H8	0.3417	1.0088	0.5654	0.025*	
C9	0.5277 (2)	1.1096 (2)	0.6400 (2)	0.0230 (5)	
H9	0.5087	1.1804	0.6249	0.028*	
C10	0.6555 (2)	1.1059 (2)	0.6987 (2)	0.0216 (5)	
H10	0.7250	1.1744	0.7240	0.026*	
C11	0.6799 (2)	1.00236 (19)	0.7197 (2)	0.0186 (4)	
H11	0.7671	1.0009	0.7606	0.022*	
C12	0.5938 (2)	0.76773 (19)	0.8711 (2)	0.0200 (4)	0.5
N12A	0.5938 (2)	0.76773 (19)	0.8711 (2)	0.0200 (4)	0.5
C13	0.5758 (2)	0.8649 (2)	0.9321 (2)	0.0188 (4)	
H13	0.5702	0.9308	0.8983	0.023*	
C14	0.5659 (2)	0.8671 (2)	1.0412 (2)	0.0217 (5)	
H14	0.5557	0.9348	1.0818	0.026*	
C15	0.5709 (2)	0.7710 (2)	1.0910 (2)	0.0239 (5)	
H15	0.5613	0.7719	1.1640	0.029*	
C16	0.5899 (2)	0.6735 (2)	1.0337 (2)	0.0217 (5)	
H16	0.5949	0.6081	1.0682	0.026*	
C17	0.6018 (2)	0.67162 (19)	0.9253 (2)	0.0166 (4)	
C18	0.6277 (2)	0.57502 (19)	0.8611 (2)	0.0161 (4)	
C19	0.6551 (2)	0.4771 (2)	0.9021 (2)	0.0209 (5)	
H19	0.6533	0.4661	0.9762	0.025*	
C20	0.6848 (3)	0.3963 (2)	0.8351 (2)	0.0249 (5)	
H20	0.7057	0.3305	0.8638	0.030*	
C21	0.6838 (3)	0.4120 (2)	0.7249 (2)	0.0246 (5)	
H21	0.7021	0.3564	0.6764	0.029*	
C22	0.6558 (2)	0.5097 (2)	0.6875 (2)	0.0200 (5)	
H22	0.6547	0.5205	0.6122	0.024*	
C23	0.7243 (2)	0.74819 (19)	0.5292 (2)	0.0171 (4)	
C24	0.7062 (2)	0.7315 (2)	0.4058 (2)	0.0219 (5)	
H24	0.6237	0.6883	0.3408	0.026*	
C25	0.8046 (3)	0.7758 (3)	0.3775 (2)	0.0288 (6)	
H25	0.7881	0.7644	0.2938	0.035*	
C26	0.9283 (3)	0.8375 (3)	0.4708 (3)	0.0322 (6)	
H26	0.9950	0.8709	0.4511	0.039*	
C27	0.9517 (2)	0.8488 (3)	0.5906 (3)	0.0283 (6)	
H27	1.0370	0.8866	0.6538	0.034*	
C28	0.8532 (2)	0.8061 (2)	0.6238 (2)	0.0198 (5)	
C29	0.8935 (2)	0.8164 (2)	0.7526 (2)	0.0198 (5)	
H29	0.9842	0.8371	0.8037	0.024*	
C30	0.8861 (2)	0.7980 (2)	0.9349 (2)	0.0191 (5)	
C31	0.9382 (2)	0.7062 (2)	0.9630 (2)	0.0254 (5)	
H31	0.9380	0.6505	0.9000	0.030*	
C32	0.9906 (3)	0.6962 (3)	1.0839 (3)	0.0332 (6)	
H32	1.0255	0.6332	1.1034	0.040*	
C33	0.9920 (3)	0.7784 (3)	1.1760 (3)	0.0371 (7)	
H33	1.0267	0.7710	1.2583	0.044*	
C34	0.9430 (3)	0.8704 (3)	1.1480 (2)	0.0330 (6)	
H34	0.9460	0.9274	1.2118	0.040*	
C35	0.8889 (2)	0.8813 (2)	1.0276 (2)	0.0247 (5)	
H35	0.8545	0.9448	1.0088	0.030*	

### Atomic displacement parameters (Å^2^)

**Table:**

	*U*^11^	*U*^22^	*U*^33^	*U*^12^	*U*^13^	*U*^23^
Ir1	0.01277 (5)	0.01203 (5)	0.01265 (5)	0.00444 (3)	0.00584 (3)	0.00369 (3)
Cl1	0.0270 (4)	0.0682 (6)	0.0609 (6)	0.0188 (4)	0.0103 (4)	−0.0109 (5)
Cl2	0.0591 (6)	0.0585 (6)	0.0485 (5)	−0.0171 (4)	0.0221 (4)	0.0061 (4)
C36	0.0333 (15)	0.0408 (17)	0.0376 (17)	0.0026 (13)	0.0178 (13)	0.0052 (13)
O1	0.0181 (8)	0.0205 (8)	0.0157 (8)	0.0045 (6)	0.0083 (6)	0.0042 (6)
N1	0.0127 (9)	0.0112 (8)	0.0112 (9)	0.0054 (7)	0.0055 (7)	0.0039 (7)
C1A	0.0127 (9)	0.0112 (8)	0.0112 (9)	0.0054 (7)	0.0055 (7)	0.0039 (7)
N2	0.0122 (9)	0.0102 (8)	0.0125 (9)	0.0036 (7)	0.0051 (7)	0.0022 (7)
C2A	0.0122 (9)	0.0102 (8)	0.0125 (9)	0.0036 (7)	0.0051 (7)	0.0022 (7)
N3	0.0155 (9)	0.0155 (9)	0.0145 (9)	0.0056 (7)	0.0059 (7)	0.0044 (7)
C1	0.0213 (11)	0.0236 (11)	0.0182 (11)	0.0057 (9)	0.0093 (9)	0.0040 (9)
N1A	0.0213 (11)	0.0236 (11)	0.0182 (11)	0.0057 (9)	0.0093 (9)	0.0040 (9)
C2	0.0213 (11)	0.0186 (11)	0.0177 (11)	0.0043 (9)	0.0091 (9)	0.0032 (9)
C3	0.0194 (11)	0.0223 (12)	0.0234 (12)	−0.0007 (9)	0.0093 (10)	0.0028 (10)
C4	0.0157 (11)	0.0313 (13)	0.0243 (13)	0.0025 (10)	0.0050 (10)	0.0053 (11)
C5	0.0163 (11)	0.0250 (12)	0.0244 (12)	0.0076 (9)	0.0063 (10)	0.0067 (10)
C6	0.0164 (10)	0.0176 (11)	0.0158 (11)	0.0044 (8)	0.0067 (9)	0.0038 (9)
C7	0.0162 (10)	0.0181 (11)	0.0152 (10)	0.0060 (8)	0.0072 (9)	0.0047 (8)
C8	0.0196 (11)	0.0219 (11)	0.0219 (12)	0.0107 (9)	0.0075 (9)	0.0066 (9)
C9	0.0267 (12)	0.0161 (11)	0.0283 (13)	0.0093 (9)	0.0125 (11)	0.0076 (10)
C10	0.0211 (11)	0.0160 (11)	0.0260 (13)	0.0043 (9)	0.0097 (10)	0.0048 (9)
C11	0.0161 (10)	0.0172 (11)	0.0211 (11)	0.0041 (8)	0.0074 (9)	0.0048 (9)
C12	0.0166 (10)	0.0215 (10)	0.0196 (11)	0.0051 (8)	0.0065 (8)	0.0039 (9)
N12A	0.0166 (10)	0.0215 (10)	0.0196 (11)	0.0051 (8)	0.0065 (8)	0.0039 (9)
C13	0.0197 (11)	0.0191 (11)	0.0207 (11)	0.0089 (9)	0.0102 (9)	0.0064 (9)
C14	0.0207 (11)	0.0263 (12)	0.0196 (12)	0.0111 (10)	0.0099 (9)	0.0016 (10)
C15	0.0241 (12)	0.0348 (14)	0.0159 (11)	0.0102 (10)	0.0110 (10)	0.0066 (10)
C16	0.0236 (12)	0.0253 (12)	0.0195 (12)	0.0084 (10)	0.0111 (10)	0.0093 (10)
C17	0.0146 (10)	0.0182 (10)	0.0173 (11)	0.0052 (8)	0.0070 (9)	0.0051 (9)
C18	0.0142 (10)	0.0156 (10)	0.0160 (10)	0.0018 (8)	0.0060 (8)	0.0031 (8)
C19	0.0251 (12)	0.0196 (11)	0.0211 (12)	0.0073 (9)	0.0117 (10)	0.0089 (9)
C20	0.0324 (13)	0.0169 (11)	0.0293 (13)	0.0111 (10)	0.0144 (11)	0.0103 (10)
C21	0.0315 (13)	0.0180 (11)	0.0278 (13)	0.0105 (10)	0.0159 (11)	0.0038 (10)
C22	0.0244 (12)	0.0170 (11)	0.0207 (12)	0.0071 (9)	0.0120 (10)	0.0036 (9)
C23	0.0203 (11)	0.0186 (10)	0.0182 (11)	0.0101 (9)	0.0105 (9)	0.0092 (9)
C24	0.0233 (12)	0.0289 (13)	0.0171 (11)	0.0125 (10)	0.0096 (10)	0.0083 (10)
C25	0.0305 (14)	0.0472 (16)	0.0221 (13)	0.0193 (12)	0.0182 (11)	0.0163 (12)
C26	0.0246 (13)	0.0537 (18)	0.0297 (14)	0.0149 (12)	0.0188 (12)	0.0184 (13)
C27	0.0192 (12)	0.0420 (15)	0.0271 (13)	0.0104 (11)	0.0121 (10)	0.0112 (12)
C28	0.0200 (11)	0.0230 (11)	0.0206 (12)	0.0100 (9)	0.0108 (9)	0.0072 (9)
C29	0.0165 (11)	0.0215 (11)	0.0205 (12)	0.0071 (9)	0.0066 (9)	0.0061 (9)
C30	0.0115 (10)	0.0251 (12)	0.0175 (11)	0.0021 (9)	0.0047 (9)	0.0065 (9)
C31	0.0187 (11)	0.0314 (13)	0.0266 (13)	0.0089 (10)	0.0085 (10)	0.0115 (11)
C32	0.0225 (13)	0.0471 (17)	0.0296 (15)	0.0105 (12)	0.0069 (11)	0.0234 (13)
C33	0.0225 (13)	0.061 (2)	0.0204 (13)	0.0014 (13)	0.0053 (11)	0.0167 (13)
C34	0.0240 (13)	0.0474 (17)	0.0177 (12)	−0.0006 (12)	0.0083 (10)	−0.0019 (12)
C35	0.0202 (12)	0.0308 (13)	0.0193 (12)	0.0033 (10)	0.0086 (10)	0.0025 (10)

### Geometric parameters (Å, º)

**Table:**

Ir1—N12A	1.997 (2)	C11—H11	0.9500
Ir1—C12	1.997 (2)	C12—C13	1.400 (3)
Ir1—N1A	2.004 (2)	C12—C17	1.418 (3)
Ir1—C1	2.004 (2)	N12A—C13	1.400 (3)
Ir1—N2	2.0302 (18)	N12A—C17	1.418 (3)
Ir1—C2A	2.0302 (18)	C13—C14	1.393 (3)
Ir1—N1	2.0424 (18)	C13—H13	0.9500
Ir1—C1A	2.0424 (18)	C14—C15	1.388 (4)
Ir1—O1	2.1409 (16)	C14—H14	0.9500
Ir1—N3	2.1551 (19)	C15—C16	1.389 (4)
Cl1—C36	1.769 (3)	C15—H15	0.9500
Cl2—C36	1.749 (3)	C16—C17	1.396 (3)
C36—H36A	0.9900	C16—H16	0.9500
C36—H36B	0.9900	C17—C18	1.467 (3)
O1—C23	1.295 (3)	C18—C19	1.394 (3)
N1—C11	1.352 (3)	C19—C20	1.377 (3)
N1—C7	1.365 (3)	C19—H19	0.9500
C1A—C11	1.352 (3)	C20—C21	1.394 (4)
C1A—C7	1.365 (3)	C20—H20	0.9500
N2—C22	1.350 (3)	C21—C22	1.377 (3)
N2—C18	1.363 (3)	C21—H21	0.9500
C2A—C22	1.350 (3)	C22—H22	0.9500
C2A—C18	1.363 (3)	C23—C24	1.422 (3)
N3—C29	1.300 (3)	C23—C28	1.433 (3)
N3—C30	1.440 (3)	C24—C25	1.380 (4)
C1—C2	1.403 (3)	C24—H24	0.9500
C1—C6	1.417 (3)	C25—C26	1.399 (4)
N1A—C2	1.403 (3)	C25—H25	0.9500
N1A—C6	1.417 (3)	C26—C27	1.365 (4)
C2—C3	1.392 (3)	C26—H26	0.9500
C2—H2	0.9500	C27—C28	1.416 (3)
C3—C4	1.396 (4)	C27—H27	0.9500
C3—H3	0.9500	C28—C29	1.435 (3)
C4—C5	1.391 (4)	C29—H29	0.9500
C4—H4	0.9500	C30—C31	1.390 (3)
C5—C6	1.398 (3)	C30—C35	1.393 (3)
C5—H5	0.9500	C31—C32	1.392 (4)
C6—C7	1.468 (3)	C31—H31	0.9500
C7—C8	1.395 (3)	C32—C33	1.387 (5)
C8—C9	1.382 (3)	C32—H32	0.9500
C8—H8	0.9500	C33—C34	1.374 (4)
C9—C10	1.395 (3)	C33—H33	0.9500
C9—H9	0.9500	C34—C35	1.391 (4)
C10—C11	1.372 (3)	C34—H34	0.9500
C10—H10	0.9500	C35—H35	0.9500
			
N12A—Ir1—N1A	87.97 (9)	C11—C10—H10	120.3
C12—Ir1—C1	87.97 (9)	C9—C10—H10	120.3
C12—Ir1—N2	80.69 (8)	C1A—C11—C10	122.4 (2)
C1—Ir1—N2	96.62 (8)	N1—C11—C10	122.4 (2)
N12A—Ir1—C2A	80.69 (8)	N1—C11—H11	118.8
N1A—Ir1—C2A	96.62 (8)	C10—C11—H11	118.8
C12—Ir1—N1	95.15 (8)	C13—C12—C17	117.2 (2)
C1—Ir1—N1	80.42 (8)	C13—C12—Ir1	128.28 (17)
N2—Ir1—N1	175.02 (7)	C17—C12—Ir1	114.53 (16)
N12A—Ir1—C1A	95.15 (8)	C13—N12A—C17	117.2 (2)
N1A—Ir1—C1A	80.42 (8)	C13—N12A—Ir1	128.28 (17)
C2A—Ir1—C1A	175.02 (7)	C17—N12A—Ir1	114.53 (16)
N12A—Ir1—O1	175.01 (7)	C14—C13—C12	121.5 (2)
C12—Ir1—O1	175.01 (7)	C14—C13—N12A	121.5 (2)
N1A—Ir1—O1	90.14 (8)	C14—C13—H13	119.3
C1—Ir1—O1	90.14 (8)	C12—C13—H13	119.3
N2—Ir1—O1	94.95 (7)	C15—C14—C13	120.4 (2)
C2A—Ir1—O1	94.95 (7)	C15—C14—H14	119.8
N1—Ir1—O1	89.08 (7)	C13—C14—H14	119.8
C1A—Ir1—O1	89.08 (7)	C14—C15—C16	119.7 (2)
N12A—Ir1—N3	94.96 (8)	C14—C15—H15	120.2
C12—Ir1—N3	94.96 (8)	C16—C15—H15	120.2
N1A—Ir1—N3	176.86 (7)	C15—C16—C17	120.1 (2)
C1—Ir1—N3	176.86 (7)	C15—C16—H16	120.0
N2—Ir1—N3	84.99 (7)	C17—C16—H16	120.0
C2A—Ir1—N3	84.99 (7)	C16—C17—C12	121.2 (2)
N1—Ir1—N3	98.16 (7)	C16—C17—N12A	121.2 (2)
C1A—Ir1—N3	98.16 (7)	C16—C17—C18	124.0 (2)
O1—Ir1—N3	87.04 (7)	C12—C17—C18	114.8 (2)
Cl2—C36—Cl1	110.91 (17)	N12A—C17—C18	114.8 (2)
Cl2—C36—H36A	109.5	C2A—C18—C19	119.9 (2)
Cl1—C36—H36A	109.5	N2—C18—C19	119.9 (2)
Cl2—C36—H36B	109.5	C2A—C18—C17	113.50 (19)
Cl1—C36—H36B	109.5	N2—C18—C17	113.50 (19)
H36A—C36—H36B	108.0	C19—C18—C17	126.5 (2)
C23—O1—Ir1	126.57 (15)	C20—C19—C18	120.1 (2)
C11—N1—C7	119.07 (19)	C20—C19—H19	120.0
C11—N1—Ir1	124.65 (15)	C18—C19—H19	120.0
C7—N1—Ir1	116.26 (15)	C19—C20—C21	119.3 (2)
C11—C1A—C7	119.07 (19)	C19—C20—H20	120.3
C11—C1A—Ir1	124.65 (15)	C21—C20—H20	120.3
C7—C1A—Ir1	116.26 (15)	C22—C21—C20	118.7 (2)
C22—N2—C18	119.81 (19)	C22—C21—H21	120.6
C22—N2—Ir1	123.55 (16)	C20—C21—H21	120.6
C18—N2—Ir1	116.30 (15)	C2A—C22—C21	122.1 (2)
C22—C2A—C18	119.81 (19)	N2—C22—C21	122.1 (2)
C22—C2A—Ir1	123.55 (16)	N2—C22—H22	119.0
C18—C2A—Ir1	116.30 (15)	C21—C22—H22	119.0
C29—N3—C30	115.83 (19)	O1—C23—C24	118.1 (2)
C29—N3—Ir1	124.64 (16)	O1—C23—C28	125.5 (2)
C30—N3—Ir1	118.36 (14)	C24—C23—C28	116.4 (2)
C2—C1—C6	116.8 (2)	C25—C24—C23	122.2 (2)
C2—C1—Ir1	128.52 (17)	C25—C24—H24	118.9
C6—C1—Ir1	114.69 (16)	C23—C24—H24	118.9
C2—N1A—C6	116.8 (2)	C24—C25—C26	120.7 (2)
C2—N1A—Ir1	128.52 (17)	C24—C25—H25	119.7
C6—N1A—Ir1	114.69 (16)	C26—C25—H25	119.7
C3—C2—C1	121.8 (2)	C27—C26—C25	118.9 (2)
C3—C2—N1A	121.8 (2)	C27—C26—H26	120.6
C3—C2—H2	119.1	C25—C26—H26	120.6
C1—C2—H2	119.1	C26—C27—C28	122.3 (2)
C2—C3—C4	120.5 (2)	C26—C27—H27	118.9
C2—C3—H3	119.8	C28—C27—H27	118.9
C4—C3—H3	119.8	C27—C28—C23	119.4 (2)
C5—C4—C3	119.3 (2)	C27—C28—C29	116.2 (2)
C5—C4—H4	120.4	C23—C28—C29	124.2 (2)
C3—C4—H4	120.4	N3—C29—C28	127.8 (2)
C4—C5—C6	120.1 (2)	N3—C29—H29	116.1
C4—C5—H5	119.9	C28—C29—H29	116.1
C6—C5—H5	119.9	C31—C30—C35	120.3 (2)
C5—C6—C1	121.6 (2)	C31—C30—N3	119.0 (2)
C5—C6—N1A	121.6 (2)	C35—C30—N3	120.6 (2)
C5—C6—C7	123.4 (2)	C30—C31—C32	119.7 (3)
C1—C6—C7	115.01 (19)	C30—C31—H31	120.2
N1A—C6—C7	115.01 (19)	C32—C31—H31	120.2
C1A—C7—C8	120.4 (2)	C33—C32—C31	120.1 (3)
N1—C7—C8	120.4 (2)	C33—C32—H32	120.0
C1A—C7—C6	113.51 (19)	C31—C32—H32	120.0
N1—C7—C6	113.51 (19)	C34—C33—C32	119.9 (3)
C8—C7—C6	126.0 (2)	C34—C33—H33	120.0
C9—C8—C7	120.2 (2)	C32—C33—H33	120.0
C9—C8—H8	119.9	C33—C34—C35	120.9 (3)
C7—C8—H8	119.9	C33—C34—H34	119.5
C8—C9—C10	118.5 (2)	C35—C34—H34	119.5
C8—C9—H9	120.7	C34—C35—C30	119.1 (2)
C10—C9—H9	120.7	C34—C35—H35	120.4
C11—C10—C9	119.4 (2)	C30—C35—H35	120.4
			
C6—C1—C2—C3	1.1 (3)	C13—N12A—C17—C16	1.0 (3)
Ir1—C1—C2—C3	−178.72 (18)	Ir1—N12A—C17—C16	−179.75 (18)
C6—N1A—C2—C3	1.1 (3)	C13—N12A—C17—C18	−176.82 (19)
Ir1—N1A—C2—C3	−178.72 (18)	Ir1—N12A—C17—C18	2.4 (2)
C1—C2—C3—C4	−1.1 (4)	C22—C2A—C18—C19	1.5 (3)
N1A—C2—C3—C4	−1.1 (4)	Ir1—C2A—C18—C19	−172.00 (17)
C2—C3—C4—C5	0.2 (4)	C22—C2A—C18—C17	178.4 (2)
C3—C4—C5—C6	0.4 (4)	Ir1—C2A—C18—C17	4.9 (2)
C4—C5—C6—C1	−0.3 (4)	C22—N2—C18—C19	1.5 (3)
C4—C5—C6—N1A	−0.3 (4)	Ir1—N2—C18—C19	−172.00 (17)
C4—C5—C6—C7	178.8 (2)	C22—N2—C18—C17	178.4 (2)
C2—C1—C6—C5	−0.5 (3)	Ir1—N2—C18—C17	4.9 (2)
Ir1—C1—C6—C5	179.43 (18)	C16—C17—C18—C2A	177.5 (2)
C2—C1—C6—C7	−179.7 (2)	N12A—C17—C18—C2A	−4.7 (3)
Ir1—C1—C6—C7	0.2 (3)	C16—C17—C18—N2	177.5 (2)
C2—N1A—C6—C5	−0.5 (3)	C12—C17—C18—N2	−4.7 (3)
Ir1—N1A—C6—C5	179.43 (18)	C16—C17—C18—C19	−5.8 (4)
C2—N1A—C6—C7	−179.7 (2)	C12—C17—C18—C19	171.9 (2)
Ir1—N1A—C6—C7	0.2 (3)	N12A—C17—C18—C19	171.9 (2)
C11—C1A—C7—C8	−1.6 (3)	C2A—C18—C19—C20	0.2 (4)
Ir1—C1A—C7—C8	176.93 (17)	N2—C18—C19—C20	0.2 (4)
C11—C1A—C7—C6	177.9 (2)	C17—C18—C19—C20	−176.3 (2)
Ir1—C1A—C7—C6	−3.6 (2)	C18—C19—C20—C21	−1.6 (4)
C11—N1—C7—C8	−1.6 (3)	C19—C20—C21—C22	1.4 (4)
Ir1—N1—C7—C8	176.93 (17)	C18—C2A—C22—C21	−1.7 (3)
C11—N1—C7—C6	177.9 (2)	Ir1—C2A—C22—C21	171.25 (19)
Ir1—N1—C7—C6	−3.6 (2)	C18—N2—C22—C21	−1.7 (3)
C5—C6—C7—C1A	−177.0 (2)	Ir1—N2—C22—C21	171.25 (19)
N1A—C6—C7—C1A	2.2 (3)	C20—C21—C22—C2A	0.3 (4)
C5—C6—C7—N1	−177.0 (2)	C20—C21—C22—N2	0.3 (4)
C1—C6—C7—N1	2.2 (3)	Ir1—O1—C23—C24	−167.35 (15)
C5—C6—C7—C8	2.4 (4)	Ir1—O1—C23—C28	14.9 (3)
C1—C6—C7—C8	−178.4 (2)	O1—C23—C24—C25	177.4 (2)
N1A—C6—C7—C8	−178.4 (2)	C28—C23—C24—C25	−4.6 (3)
C1A—C7—C8—C9	2.1 (4)	C23—C24—C25—C26	1.5 (4)
N1—C7—C8—C9	2.1 (4)	C24—C25—C26—C27	2.8 (4)
C6—C7—C8—C9	−177.3 (2)	C25—C26—C27—C28	−3.7 (4)
C7—C8—C9—C10	−1.2 (4)	C26—C27—C28—C23	0.5 (4)
C8—C9—C10—C11	−0.2 (4)	C26—C27—C28—C29	175.6 (3)
C7—C1A—C11—C10	0.2 (3)	O1—C23—C28—C27	−178.6 (2)
Ir1—C1A—C11—C10	−178.21 (18)	C24—C23—C28—C27	3.6 (3)
C7—N1—C11—C10	0.2 (3)	O1—C23—C28—C29	6.7 (4)
Ir1—N1—C11—C10	−178.21 (18)	C24—C23—C28—C29	−171.1 (2)
C9—C10—C11—C1A	0.8 (4)	C30—N3—C29—C28	170.2 (2)
C9—C10—C11—N1	0.8 (4)	Ir1—N3—C29—C28	2.8 (3)
C17—C12—C13—C14	−0.1 (3)	C27—C28—C29—N3	168.6 (2)
Ir1—C12—C13—C14	−179.18 (18)	C23—C28—C29—N3	−16.6 (4)
C17—N12A—C13—C14	−0.1 (3)	C29—N3—C30—C31	−66.6 (3)
Ir1—N12A—C13—C14	−179.18 (18)	Ir1—N3—C30—C31	101.6 (2)
C12—C13—C14—C15	−1.4 (4)	C29—N3—C30—C35	117.9 (2)
N12A—C13—C14—C15	−1.4 (4)	Ir1—N3—C30—C35	−73.9 (2)
C13—C14—C15—C16	2.0 (4)	C35—C30—C31—C32	1.5 (4)
C14—C15—C16—C17	−1.1 (4)	N3—C30—C31—C32	−174.0 (2)
C15—C16—C17—C12	−0.5 (4)	C30—C31—C32—C33	−0.7 (4)
C15—C16—C17—N12A	−0.5 (4)	C31—C32—C33—C34	−0.8 (4)
C15—C16—C17—C18	177.2 (2)	C32—C33—C34—C35	1.5 (4)
C13—C12—C17—C16	1.0 (3)	C33—C34—C35—C30	−0.6 (4)
Ir1—C12—C17—C16	−179.75 (18)	C31—C30—C35—C34	−0.9 (4)
C13—C12—C17—C18	−176.82 (19)	N3—C30—C35—C34	174.6 (2)
Ir1—C12—C17—C18	2.4 (2)		

### Hydrogen-bond geometry (Å, º)

*Cg*2 and *Cg*3 are the centroids of the C1–C6 and C12–C17 rings, respectively.

**Table:**

*D*—H···*A*	*D*—H	H···*A*	*D*···*A*	*D*—H···*A*
C22—H22···O1	0.95	2.54	3.132 (3)	121
C21—H21···*Cg*2^i^	0.95	2.90	3.658 (3)	138
C36—H36*A*···*Cg*2	0.99	2.62	3.444 (4)	140
C36—H36*B*···*Cg*3	0.99	2.59	3.498 (4)	153

Symmetry code: (i) −*x*+1, −*y*+1, −*z*+1.
